# Feather bacterial load shapes the trade-off between preening and immunity in pigeons

**DOI:** 10.1186/s12862-015-0338-9

**Published:** 2015-04-07

**Authors:** Sarah Leclaire, Gábor Árpád Czirják, Abdessalem Hammouda, Julien Gasparini

**Affiliations:** Sorbonnes Universités, UPMC Université Paris 06, Institut d’Ecologie et des Sciences de l’Environnement de Paris, Paris, F-75005 France; CEFE-CNRS, UMR5175, Centre d’Ecologie Fonctionnelle et Evolutive, 1919 Rte de Mende, Montpellier, 34293 France; Department of Wildlife Diseases, Leibniz Institute for Zoo and Wildlife Research, Alfred-Kowalke-Straße 17, Berlin, 10315 Germany; Département des Sciences de la Vie, Faculté des Sciences de Gabès, Cité Erriadh, Zrig 6072, Gabès, Tunisia

**Keywords:** Microbiota, PHA, Bacterial killing ability, Immune system, Pigeons, *Columba livia*

## Abstract

**Background:**

Complex communities of bacteria inhabit the feathers of all birds. Under normal conditions, individuals maintain a healthy state by defending themselves against these potential invaders by preening. The immune system is only triggered when bacteria gain access into the body. Preening is, however, costly and may trade-off with investment in the immune system. To shed light on how birds balance the trade-off between immunity and preen secretions when facing high or low feather bacterial load, we experimentally manipulated feather bacteria load of feral pigeons (*Columba livia*), and investigated the effects on immune defenses.

**Results:**

Birds facing high feather bacterial load had lower immune response to PHA skin-swelling test (a measure of induced pro-inflammatory capacity) than controls, while birds facing low feather bacterial load had higher blood bacterial killing ability (a measure of the capacity to eliminate bacterial pathogens) than controls. No other components of the immune system (*i.e.*, hemagglutination and hemolysis capacity of plasma, primary and secondary responses to KLH and quantity of blood parasites) were found to be affected by feather bacterial load.

**Conclusion:**

Pigeons had previously been shown to adjust preening to feather bacterial load. The decrease in the energetically costly inflammatory response of birds experiencing high bacterial load suggests a trade-off between investment in preen secretion and immunity and reinforces the idea that feather microbiota may have a strong impact on the ecology and evolution of the avian host.

## Background

Microorganisms constitute more than half of the total biomass of Earth; however, we have just made the first steps in discovering and measuring the diversity, abundance and function of these microbial ecosystems [1-4]. Macroorganisms live, therefore, with a pool of microorganisms, being in close contact and continuously interacting with them. Some of the microorganisms are beneficial and mutualistic relationships have evolved with the host, while others can have detrimental effects on the host [5]. To defend themselves against these potential parasites and maintain their health status, host organisms have evolved a complex system of behavioral, mechanical and chemical defenses [6-9], the immune system being of the utmost importance.

In birds, some bacteria inhabiting the feathers are detrimental to the bird by degrading keratin [10], thus altering thermoregulation, flight and ultimately fitness [11-13]. To maintain feather condition, birds have evolved several anti-bacterial defenses [14], including the deposition of preen secretions onto feathers (*i.e.*, preening). Preening may have antibacterial effects either by the direct activity of antibacterial compounds [15], by antibacterial substances secreted by the uropygial gland-associated symbiotic bacteria [16] or by forming a mechanical barrier between the bacteria and the feather surface [17]. Recently it has been shown that preen secretion production and preening frequency are adjusted to feather bacterial load, suggesting that they are energetically costly inducible antibacterial defenses [18,19]. Preen secretions are composed mainly of oily substances [20] which probably require considerable energy in their anabolism. In addition, preening is an energetically costly behavior [21] that takes a substantial portion of a bird’s time budget [22]. Preening and preen secretion therefore probably compete with other costly life-history traits. In particular, investment in preen gland associated traits (*e.g.*, volume of the secretions and preening behavior) have been suggested to trade-off with investment in immune defense. Accordingly, immune-challenged or experimentally-infected tawny owls (*Strix aluco*) and house sparrows (*Passer domesticus*) develop smaller preen glands [23-25], while apapanes (*Himatione sanguinea*) infected with plasmodium preen less frequently [26].

Although feather bacteria load is essentially controlled by preen secretions, some feather bacteria can gain access into the tissue under specific conditions and become opportunistic pathogens, as demonstrated for some usually benign skin bacteria in humans [27]. Therefore, selection may favor maintaining an appropriate level of immune defense - the final line of defense against parasites - when facing high feather bacterial load. How birds invest in the energetically competing defenses that are preen secretions and the immune system when facing varying degree of feather bacteria load is, however, unknown. In a previous study, we have shown that preen secretion quantity and preening frequency increased with experimentally increased feather bacterial load in captive feral pigeons (*Columba livia*) [18]. Therefore, to shed light on how birds balance the trade-off between immunity and preen secretions when facing high or low feather bacterial loads, we investigated the effect of feather bacteria load on immune defense in the same experimental pigeons. Given the potential trade-off between preen secretion and immunity, birds experiencing high bacterial load are expected to have decreased overall immune response. However, they are expected also to maintain immune defenses against microparasites, against which the first line of defense is mediated by constitutive innate immunity, a mixture of humoral (*e.g.*, natural antibodies, complement protein, antimicrobial peptides) and cellular components (*e.g.*, macrophages, heterophils, eosinophils and thrombocytes) [28].

Providing a broad characterization of the immune system of vertebrates is a non-trivial challenge in immunoecological studies [29,30], and simultaneous measurements of multiple immune parameters are necessary. Here we used five measures to assess the immunity of pigeons: (1) the response to phytohemagglutinin (PHA), a measure of induced pro-inflammatory capacity [31]; (2) the hemagglutination and the (3) hemolysis capacity of plasma, a measure of the natural antibody and complement levels, respectively [32]; (4) the bactericidal capacity of whole blood, a measure of the capacity to eliminate bacterial pathogens [33]; and (5) the response against keyhole limpet hemocyanin (KLH), a measure of induced humoral immune response. Finally, because variation in immunity is usually associated with increased susceptibility to pathogens, we measured the intensity of blood parasite infection.

## Results

BACT- birds had lower bacterial load on feathers than control (CO) birds, while BACT+ birds had higher bacterial load than CO birds (F_2,64_ = 29.89, P < 0.0001; CO vs. BACT+: P_adj_ < 0.0001 and CO vs. BACT-: P_adj_ = 0.037; see Figure one in Leclaire et al. [18]). Body condition did not differ among treatments (F_2,73_ = 1.56, P = 0.22).

**Figure 1:**
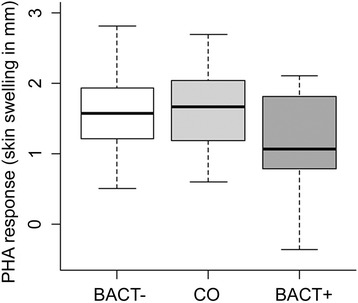
**Tukey boxplot of PHA response (skin swelling of the patagium in mm) in BACT- CO and BACT+ birds.**

PHA response varied with treatment (F_2,60_ = 4.16, P = 0.020, n = 64, Figure 1). BACT+ birds had lower PHA response than BACT- and CO birds (BACT+ vs. BACT-: P_adj_ = 0.043; BACT+ vs. CO: P_adj_ = 0.034; BACT- vs. CO: P_adj_ = 1.00; Figure 1). PHA response tended to increase with wing length (F_1,60_ = 3.98, P = 0.051) but it did not depend upon sex (F_1,58_ = 0.00, P = 0.94) or morph (F_1,59_ = 2.38, P = 0.13).

**Figure 2:**
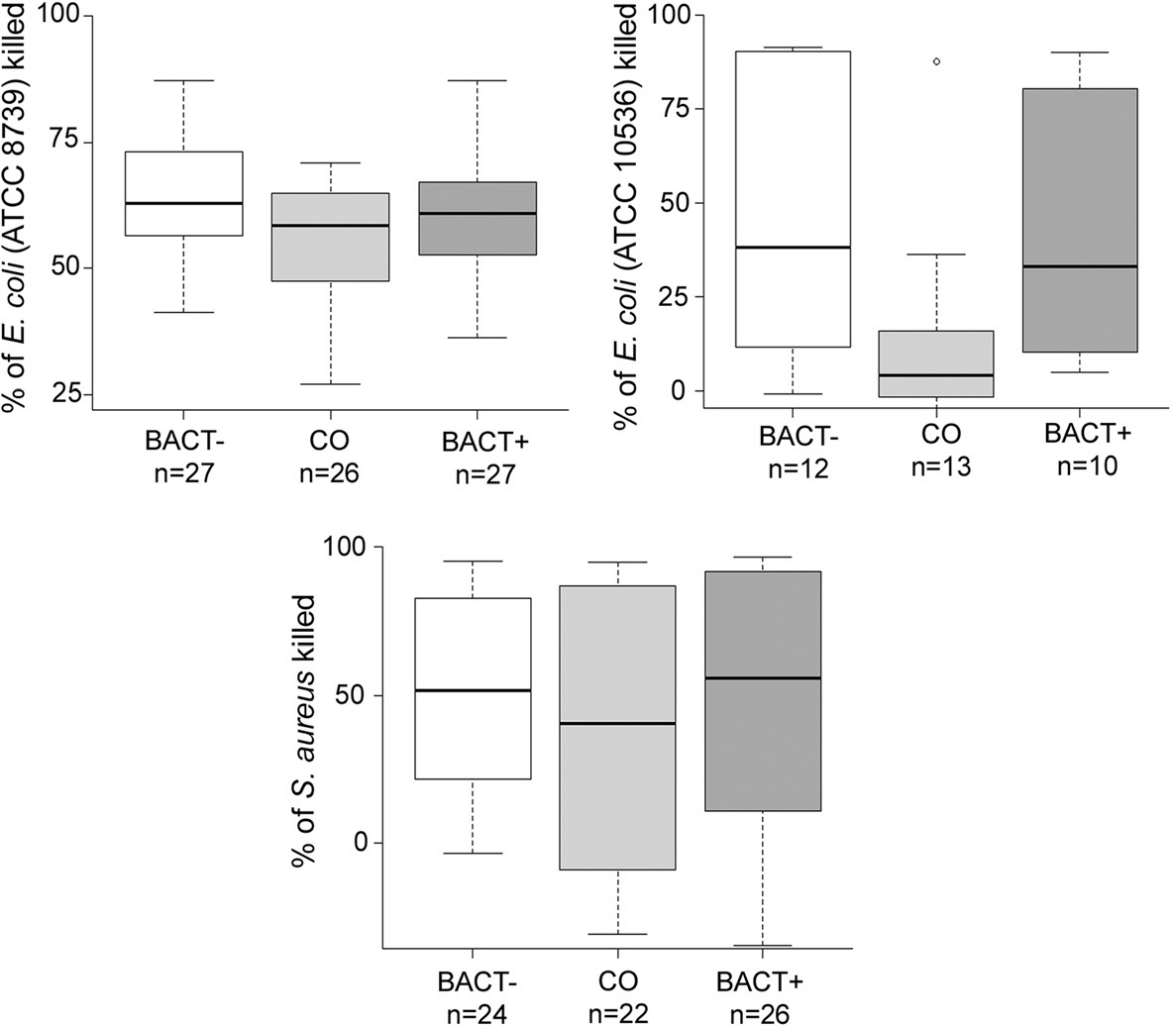
**Tukey boxplot of whole blood bacterial killing ability against** ***E. coli*** **ATCC 8739,** ***E. coli*** **ATCC 10536 and** ***S. aureus*** **in BACT-, CO and BACT+ birds.**

BACT+ and BACT- birds had higher bacterial killing ability (BKA) than CO birds for all three bacteria strains tested (*E. coli* ATCC 8739, *E. coli* ATCC 10536 and *S. aureus* ATCC 6538; Figure 2). However, differences in BKA between treatments was significant only for the two strains of *E. coli* (ATCC 8739: F_2,77_ = 3.40, P = 0.039, n = 80, and ATCC 10536: F_2,32_ = 3.54, P = 0.041, n = 35; Figure 2), while it was not significant for *S. aureus* (F_2,69_ = 0.85, P = 0.43, n = 72; Figure 2). BACT- birds had significantly higher BKA against *E. coli* than CO birds (*E. coli* ATCC 8739: P_adj_ = 0.029; *E. coli* ATCC 10536: P_adj_ = 0.046), while BACT+ birds had intermediate BKA against *E. coli* that did not differ with CO or BACT- birds (*E. coli* ATCC 8739: BACT+ vs. BACT-: P_adj_ = 0.42; BACT+ vs. CO: P_adj_ = 0.37; *E. coli* ATCC 10536: BACT+ vs. BACT-: P_adj_ = 0.92; BACT+ vs. CO: P_adj_ = 0.14). BKA did not depend upon sex (all Ps > 0.25) or color morph (*E. coli* ATCC 8739: F_1,76_ = 3.65, P = 0.060, *E. coli* ATCC 10536: F_1,31_ = 0.01, P = 0.92, *S. aureus* ATCC 6538: F_1,70_ = 0.15, P = 0.70).

Hemagglutination and hemolysis were not correlated with one another (r = 0.15, P = 0.19). Agglutination and lysis were unrelated to treatment (agglutination titer: BACT-: 7.17 ± 0.36, CO: 7.12 ± 0.42, BACT+: 6.59 ± 0.44, F_2,76_ = 0.88, P = 0.42, and lysis titer: BACT-: 3.54 ± 0.12, CO: 3.60 ± 0.11, BACT+: 3.41 ± 0.11, F_2,71.3_ = 0.74, P = 0.48). Agglutination was unrelated to sex (F_1,77_ = 2.88, P = 0.09; titer in males: 6.51 ± 0.33, titer in females: 7.35 ± 0.32), while lysis was higher in males than females (F_1,77_ = 5.50, P = 0.022; titer: 3.68 ± 0.08 and 3.37 ± 0.09 respectively). Agglutination and lysis did not vary with color morph (F_1,76.2_ = 2.65, P = 0.11 and F_1,75.5_ = 0.55, P = 0.46).

**Figure 3:**
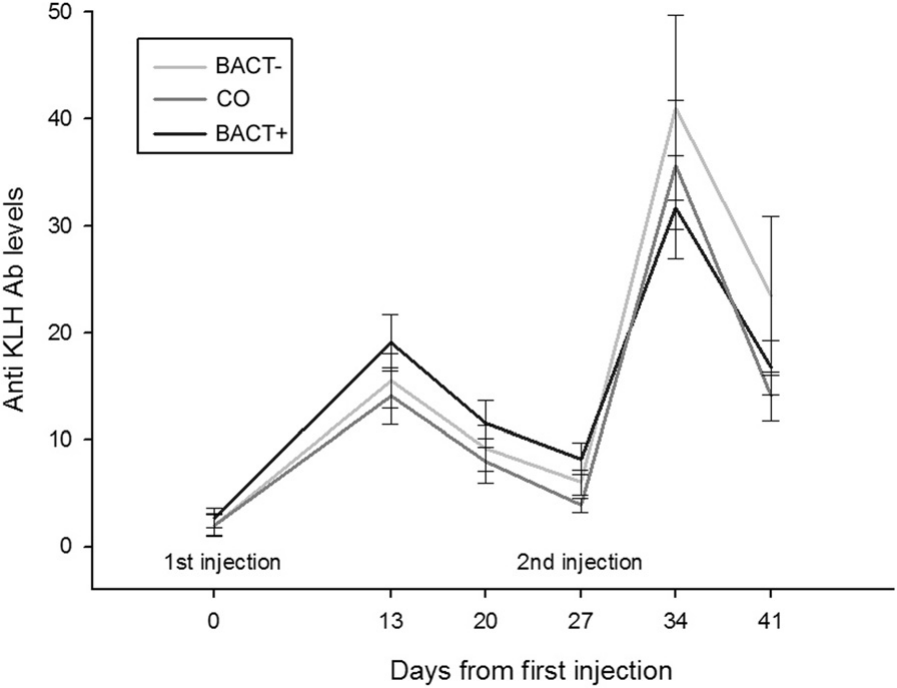
**Anti-KLH antibody levels in plasma of BACT-, CO and BACT+ birds, over the days after KLH first and second injection.**

Primary and secondary responses to KLH challenge were unrelated to treatment (F_2,74.4_ = 1.17, P = 0.32 and F_2,77.3_ = 0.39, P = 0.68; Figure 3), sex (F_2,75.3_ = 0.23, P = 0.63 and F_1,77.6_ = 0.18, P = 0.67) and color morph (F_2,75.1_ = 0.01, P = 0.91 and F_2,76.8_ = 0.04, P = 0.84).

Captivity tended to decrease blood parasite load (paired *t*-test: t_72_ =-1.9, P = 0.06). The change in blood parasite load between day 0 and day 49 of treatment was not different between BACT-, BACT+ and CO birds (F_2,70_ = 0.69, P = 0.51; BACT-: 0 ± 9 parasites; CO:-15 ± 6 parasites; BACT+:-7 ± 11 parasites). It did not vary with sex (F_1,71_ = 0.01, P = 0.94) and color morph (F_1,71_ = 0.86, P = 0.36).

## Discussion

In this study, we investigated how pigeons invest in the immune system when facing high or low feather bacterial load. Birds with increased bacterial load on plumage had lower response to PHA skin swelling test compared to control birds and birds with decreased bacterial load. PHA injection generates acute inflammation [34,35], a known energetically costly process [36], which is traded-off with various life-history traits, such as molt, breeding or growth [37,38]. In a previous study, we have found that pigeons with increased feather bacterial load invested more in behavioral and chemical defenses against feather bacteria than control birds [18]. Investment in these antibacterial defenses has been suggested to be energetically costly [25], and birds with high bacteria load on their plumage, investing more in preen secretions and preening [18], may not be able to sustain the costs of PHA-induced inflammation. Our result is, therefore, consistent with a trade-off between immunity and preening in birds [25,23]. It also suggests that birds facing high bacterial load preferentially invest in preen secretions – the first line of defense against feather bacteria – rather than in costly inflammatory processes.

The PHA response is the only immune process that varied with increased bacterial load. The bactericidal capacity of whole blood did not differ between birds with increased feather bacterial load and controls. This assay estimates the capacity of the blood to rapidly thwart a potential bacterial pathogen. This activity is a combination of several mechanisms of the innate immune system, including phagocytosis and the activities of humoral proteins such as natural antibodies, complement, antibacterial enzymes and peptides [29,33]. Thus, it is considered as the most general and integrative *in vitro* measurement of the constituent elements of the innate immune system [29]. Bactericidal activity of blood has been shown to be positively correlated with experimental or natural infection in several species [39-41]. Feather bacteria can gain entry into damaged skin and can potentially pass into the body through the gastrointestinal barrier once ingested during preening activities [42]. Birds facing high feather bacterial load may therefore be more at risk of infection by feather bacteria and may need to maintain investment in immune components against microparasites measured by the blood bactericidal activity test. Further studies are needed to determine whether pigeons with experimentally increased feather bacterial load do face higher bacterial ingestion and entry. Non-exclusively, pigeons facing high bacterial load may maintain blood bactericidal activity while decreasing PHA-induced response, because the later is more costly and thus the first to be traded-off against preen secretion.

In contrast, birds with decreased bacterial load had higher bacterial killing ability against *E. coli* than control birds. Birds with decreased bacteria load on plumage may save energy from decreased preening frequency and/or investment in preen secretion [18] and therefore may be able to invest more in these components of the innate immunity.

We did not find evidence for effects of feather bacterial load on acquired immunity in pigeons. Experimental pigeons injected with KLH, a novel antigen, did not vary the primary and secondary antibody response (*i.e.*, the capacity to recognize and make antibodies for a novel antigen and the capacity to respond to previously encountered antigen, respectively). We also did not find evidence from the hemagglutination-hemolysis assay for effects of feather bacterial load on the levels of natural antibodies (NABs) and complement. NABs are part of the innate immune system and delay pathogen replication until the developing acquired humoral and cellular immune responses clear the infection [43]. NABs are thought to be relatively insensitive to short-term changes in environmental conditions [43] and have been shown to be unaffected by experimental infections or food limitation [32,39,44-46]. Our results are in line with these studies showing that non-specific inflammation (for example, the *in vivo* PHA response) is more sensitive to changes in host condition and/or workload than are antibody responses. For example, in pied flycatchers (*Ficedula hypoleuca*), females with experimentally-reduced clutch size have lower response to PHA [47], but similar antibody responses as controls [48].

## Conclusions

In conclusion, our study suggests that increased feather bacterial load in pigeons negatively affected the components of the induced innate immune response involved in PHA-induced inflammation, while decreased feather bacterial load positively affected the components associated with the constitutive innate immune system that are measured by the bacterial killing ability test. These results are consistent with a trade-off between investment in preen secretion and immunity. No other components of the immune system were, however, found to be affected by manipulation of feather bacterial load. Whether these components were maintained to protect against feather bacteria that may enter the body or not sufficiently costly to be traded-off with preen secretion requires further study. More generally, this study reinforces the idea that feathers microbiota may have a strong impact on ecology and evolution of the avian host.

## Methods

### Experimental design

This study was carried out simultaneously with the one described in Leclaire *et al.* [18]. In March-May 2013, 80 feral pigeons (43 females and 37 males) were captured at different locations in Paris, France. They were kept in 6 outdoor aviaries at the CEREEP field station (Centre de recherche en Ecologie Expérimentale et Prédictive – Ecotron Ile-de-France, UMS 3194, Saint Pierre lès Nemours, France) in similar conditions and fed *ad libitum* with a mix of maize, wheat and peas, and mineral supplements. Birds were kept in captivity for ca. 2 months for acclimation to obtain naturally representative pigeon physiology and behavior. After acclimation, birds were assigned to treatments (BACT-: decreased feather bacterial load, BACT+: increased feather bacterial load and CO: control treatment), and they were weighed to the nearest g, wing length was measured to the nearest mm, and melanin-based color morph was recorded. Feral pigeons display a continuous variation in eumelanin-based coloration from white to black, which can be divided by human eye in 5 main groups: (1) white or almost white pigeons, (2) “blue bar” (gray mantle with two dark spots), (3) “checker” (a checked mantle with two dark wing bars), (4) “t-pattern” (a dark mantle with small graymarks), and (5) “spread” (a completely melanic plumage) [49]. This pattern correlates with differences in several life-history traits [50,51]. Therefore, we equally distributed eumelanin-based coloration of pigeons among treatments (Kruskall-Wallis test: H_*3*_ = 0.65, P = 0.89). We did the same for body mass (linear model: F_2,77_ = 0.45, P = 0.64) and body condition (measured as the residual of a regression between body mass and wing length; linear model: F_2,77_ = 1.10, P = 0.34). Birds were weighed at day 15, day 28 day 42, day 56 and day 70 after onset of treatment.

In the BACT- treatment, birds from 2 aviaries (n = 14 females and 13 males) were sprayed twice a week with 0.02% chlorhexidine (Hibitane Irrigation®, MSD) in saline solution. Chlorhexidine is an antiseptic, frequently used as a topical antiseptic skin scrub and topical disinfectant of wounds in hospitals and veterinary clinics. In the CO treatment, birds from 2 aviaries (n = 14 females and 12 males) were sprayed twice a week with saline solution. In the BACT+ treatment, birds from 2 aviaries (n = 15 females and 12 males) were sprayed twice a week with freshly cultivated bacteria in saline solution. Freshly cultivated bacteria came from feather bacteria sampled from Parisian feral pigeons and cultivated on Tryptic Soy Agar (TSA) plates and feather meal agar (FMA) plates [52]. TSA allows the growth of both keratinolytic and non-keratinolytic bacteria, while FMA allows the growth of keratinolytic bacteria only. We used both agar media to ensure the inoculation of keratinolytic bacteria in BACT+ birds. Each day of treatment, a total of 1.5 liters of solution per aviary was used to spray birds. Birds of the same aviary got the same treatment to avoid potential transmission of the treatment between birds by social interactions.

We checked the effect of treatment on feather bacterial load by cultivating feather bacteria on whole flora agar slides (plate count agar + triphenyltetrazolium chloride + neutralizing dip slides; VWR BDH Prolabo), every fortnight for 2.5 months (n = 6 control date). Slides were pressed for 10 s onto the back feathers of 4 random birds of each treatment and then incubated for 24–48 h at 37°C. Feather bacterial load was expressed as the number of bacterial colonies per slide.

### PHA skin swelling test

Three weeks after onset of treatment, 71 birds were challenged with PHA injection according to the method described by Smits *et al.* [53]. The right wing-web of each bird was injected with 0.1 ml of a 5 mg/ml PHA-P solution (Sigma L8754, St Louis, MO, USA) diluted in phosphate-buffered saline. Seven birds were excluded from the analyses as a drop of PHA solution poured out of the injection site. Wing web thickness was measured with a pressure-sensitive spessimeter (Mitutoyo, Tokyo, Japan), just before injection and 24 h after injection to assess the intensity of the immune response. To reduce measurement errors, all measurements were made 3 times in a row and by a single observer. Wing-web swelling was calculated as thickness (mean of three successive measures) of the web 24 h after injection minus thickness (mean of three successive measures) prior to injection.

### Hemagglutination-hemolysis assay

To estimate the levels of circulating natural antibodies and complement, we used the procedure developed by Matson *et al.* [32] and modified by Møller and Haussy [54]. The agglutination part of the assay estimates the interaction between natural antibodies and rabbit erythrocyte antigens. The lysis part of the assay estimates the action of complement from the amount of haemoglobin released from the lysis of rabbit erythrocytes. The quantification of agglutination and lysis is determined by serial dilution. Plates were scanned to visually determine the point when the agglutination or lysis reaction has stopped. Red blood cells were considered non-agglutinated when a red point was observed at the bottom of the well, and agglutinated when red blood cells were diffused. Red blood cells were considered lysed when the wells appeared ochre and shiny. The test was conducted 4 weeks after onset of treatment. Blood samples were collected from the alar vein with a 1-ml syringe and a sterile 26-gauge needle.

### Bactericidal capacity of whole blood

In order to assess the function of the constitutive innate immunity, we measured the bactericidal capacity of the whole blood against different bacterial strains: *Escherichia coli* ATCC 8739, *E. coli* ATCC 10536 and *Staphylococcus aureus* ATCC 6538. *E. coli* ATCC 8739 is mainly killed by the complement system [55,56], while *E. coli* ATCC 10536 and *S. aureus* ATCC 6538 are mainly killed by phagocytosis and require the presence of natural antibodies for opsonization [56,57].

*E. coli* ATCC 8739 *and S. aureus* were reconstituted from lyophilized pellets in 200 ml Tryptic Soy Broth (TSB), while the *E. coli* ATCC 10536 was kept as pure culture at -80°C in glycerol. Bacteria were grown for 24 h at 30°C with constant agitation. The solution was then centrifuged and the bacterial pellet was diluted in phosphate-buffered saline. From this stock solution, we prepared a working solution (*E. coli* ATCC 8739 and *E. coli* ATCC 10536: 10^4^ colony forming units; *S. aureus*: 10^7^ colony forming units).

The test with *E. coli* ATCC 8739 was performed 6 weeks after the onset of treatment, while the tests with *E. coli* ATCC 10536 and *S. aureus* were realized 13 weeks after the onset of treatment. Just before the tests, ca. 30 μl of blood was taken from the alar vein with sterile 26-gauge needle and transferred to sterile tubes. Within one hour, 30 μl of 1/3 diluted blood for *E. coli* ATCC 8739 test and 35 μl of fresh blood for *E. coli* ATCC 10536 and *S. aureus* tests was added to 270 μl CO_2_-independent medium with 4 mM L-glutamine (#G7513, Sigma Aldrich). 30 μl of bacteria working solution was then added. The mixture was incubated at 37°C for 30 min. Then 1.5 ml TSB was added and the mixture was incubated at 37°C under constant agitation for 12 h. Optical density at 600 nm was read using a CO 8000 Biowave cell density meter (WPA, Biochrom Ltd., Cambridge, UK). Optical density values before the 12 h incubation were used as references. Due to time constraint, the test with *E coli* ATCC 10536 was carried out on 45 birds only (12 BACT- birds, 13 CO birds, and 10 BACT+ birds).

### Primary and secondary adaptive immunity

KLH is a non-pathogenic protein antigen that is highly immunogenic, and that pigeons never encountered in the wild. Seven weeks after onset of the experiment, birds were injected subcutaneously in the lower-neck region with 50 μl of 10 mg/ml KLH. At day 0, 13, 20 and 27 after first injection, blood samples were collected in order to measure the primary antibody humoral immune response against KLH. At day 27 after first injection, a second dose of KLH was injected to measure secondary immune response. Blood was then collected at days 34 and 41 after first injection to measure the secondary antibody humoral immune response against KLH. Anti-KLH antibody (Ab) levels in plasma were assessed using a previously described method [51]. A panel of samples was used to estimate the repeatability between Ab values of the same samples between plates (between-plate repeatability: CV = 15%) as well as within plates (Pearson correlation: r = 0.99, P < 0.0001, n = 14), showing that this method is reliable for the measurement of anti-KLH Ab levels. As a standard, a mixture of several pigeon samples was measured in serial dilutions to calculate a relative Ab concentration after calibrations with this standard. This relative concentration of anti-KLH Ab was log transformed and named ‘anti-KLH Ab level’ throughout the study.

### Blood parasite load

Intensity of haemosporidian parasites (*Haemoproteus spp., Plasmodium spp.*) was measured from blood smears at onset of the experiment and after 7 weeks of treatment. Slides were fixed with methanol for 5 minutes and stained with 20% Giemsa for 45 minutes. Slides were observed microscopically under 400× and 1000× magnification. Extracellular parasites have a low prevalence, so that we focused on intracellular haemosporidian parasites (*Haemoproteus* spp. and *Plasmodium* spp.) which are responsible for the avian malaria disease. Parasite intensity was calculated as the number of infested red blood cells amongst 10000 red blood cells from different microscopic fields forming a monolayer.

### Statistical analyses

All statistical tests were conducted with SAS, version 9.2 [58]. To test whether PHA response and bactericidal capacity of whole blood depended on treatment, we used linear models (proc GLM in SAS) including treatment, sex, color morph and all two-way interactions as explanatory factors. Right wing length was included as a covariate in the PHA response model.

To test whether lyses (log-transformed) and agglutination (log-transformed) depended on treatment, we used liner mixed model (proc MIXED in SAS) including treatment, sex, color morph and all two-way interactions as explanatory factors, and plate identity as a random factor.

Primary response to KLH was tested using linear mixed models with anti-KLH Ab levels (log-transformed) at day 13, 20 and 27 after first injection as the dependent variable, and treatment, sex, color morph and all two-way interactions as explanatory factors. Days after first injection and log-transformed anti-KLH Ab levels at first injection were included as covariates, and bird identity and plate identity as random factors. Secondary response to KLH (*i.e.* anti-KLH Ab levels at day 34 and 41 after first injection) was tested using the same linear models except that day after second injection and log-transformed anti-KLH Ab levels at second injection were the covariates.

To test whether blood parasite load depended on treatment, we used a linear model with difference in blood parasite load between day 0 and day 49 of treatment as the dependent variable, and treatment, sex, color morph and all two-way interactions as explanatory factors.

We used 2-tailed type 3 tests for fixed effects with significance level set at α = 0.05. Non-significant terms were backward dropped using a stepwise elimination procedure. When the minimal model was obtained, each removed term was then put back into the minimal model to assess the level of nonsignificance. None of the two way-interactions were significant and we decided not to report their P values. When the treatment effect was significant, we used Tukey’s tests to determine the treatments that significantly differ from the others. Values are expressed as means ± SE throughout.

## Availability of supporting data

The data set supporting the results of this article is available in the Dryad repository, doi:10.5061/dryad.mm36n (http://doi.org/10.5061/dryad.mm36n).
